# Plant Diversity and Abundance Response to the Effects of Soil Properties and Growth Forms in Grasslands

**DOI:** 10.3390/plants15121895

**Published:** 2026-06-18

**Authors:** Mamokete N. V. Dingaan, Moseketsi V. Mochesane

**Affiliations:** Department of Life and Consumer Sciences, University of South Africa, Private Bag X6, Florida, Johannesburg 1710, South Africa

**Keywords:** land-use types, plant cover, plant density, plant–soil interactions

## Abstract

Biodiversity strongly influences key ecosystem processes. However, with ongoing global biodiversity loss, vital ecosystem services are likely to be negatively affected. Studies that monitor biodiversity are thus crucial, especially when performed in relation to environmental conditions. Our study investigated how species diversity and abundance were influenced by soil properties, in association with plant growth forms (i.e., grasses, forbs, and shrubs). The study was conducted across two land-use types: three protected areas (nature reserves) and adjacent unprotected areas. Plant diversity was measured as species richness, and plant abundance was measured as plant density and cover. We used simple and multiple regression analyses, as well as detrended correspondence analysis (DCA) and redundancy analysis (RDA), to elucidate relationships among species richness, plant abundance, growth forms, and soil nutrients. Both species richness and plant abundance were mostly positively associated with soil nutrients across both land-use types. The response was more robust and varied when species were partitioned into growth forms. Growth forms were strong predictors of species richness and abundance across both land-use types, whereas the effects of soil properties were relatively weaker. When growth forms and soil properties were considered jointly, their combined effects strongly predicted species richness and abundance.

## 1. Introduction

Grasslands are among the most extensive terrestrial ecosystems, covering approximately 40% of the global land area, yet they are among the most endangered [1,2]. As they occur on highly productive lands rich with soil nutrients, grasslands have consequently undergone extensive human-induced transformation for activities such as crop production and livestock grazing [2]. Although land-use practices are essential for providing humans with natural resources and ecosystem services, much of the conversion of natural landscapes for human consumption has had negative consequences, including habitat fragmentation and biodiversity loss [3]. Effects of land-use (change) on ecosystem functioning have been well-documented and are projected to induce further biodiversity losses in the future, especially in grassland ecosystems [4]. The decline in biodiversity is of great ecological concern because it significantly affects many ecosystem processes, including resource capture, biomass production, decomposition, and nutrient cycling [5].

Plant diversity in grasslands, specifically at the local scale, is maintained by ecological processes that include species interactions and environmental conditions [6]. One of the major forms of species interactions is competition for environmental resources. According to the niche differentiation hypothesis, different species are adapted to different environmental conditions. They may coexist by occupying different niches (niche differentiation), thereby reducing interspecific competition and possibly avoiding competitive exclusion [7,8]. The competitive interactions are informed by the resource requirements of individual species [9] and by the resource utilisation strategies of the different growth forms within a community [10]. Growth forms are regarded as a basic representation of plant functional types; the latter are defined as groups of species that respond similarly to the environment and may similarly contribute to ecosystem processes [11]. Furthermore, growth forms are regarded as plant morphological adaptations to the environment [12], and they exhibit specific resource utilisation strategies linked to water and nutrient uptake, and light interception [10]. As such, growth forms differ in their ecological preferences and may respond differently to the environment [12]. Owing to differences in their specific morphologic traits and divergent responses to the same environmental conditions, growth forms can modulate resource partitioning strategies and interspecific competition avoidance within communities [13].

The availability of environmental resources that regulate plant growth, which include soil nutrients, water, and light, strongly impacts plant diversity at the local scale [14]. Among the nutrients that can potentially limit plant diversity are nitrogen, phosphorus, and potassium [15,16,17,18,19]. The response of species diversity to nutrient availability can be either positive or negative [20], but it is commonly reported to be hump-shaped [21]. Soil nutrients, especially fluctuations in their supply rates, also have a major influence on species abundance [9], and abundance can be measured in various ways, including plant density and cover (e.g., [22]).

Species diversity and abundance (i.e., density and cover) are interrelated and may influence each other. The influence of diversity on species density is primarily driven by complementarity in resource use among species. According to Marquard et al. [23], an increase in species richness increases interspecific (as opposed to intraspecific) interactions among neighbouring plant individuals because resident intraspecific individuals are replaced by newly establishing interspecific ones. This increases the likelihood that complementarity in resource utilisation (niche differentiation) will be enhanced [24], and that individual plants may respond to increased nutrient availability and niche space by increasing in density [23]. Consequently, the change in density may affect competitive interactions between species, which, in turn, is mediated by differences in growth forms [25].

The influence of plant density on diversity is driven by immigration and species’ competitive ability (i.e., regional and local processes, respectively) [26]. Plants compete for resources and for space. When the density of poorly or moderately competitive species increases, plant diversity increases as well, however, when the density of highly competitive species increases, their density increase may result in competitive exclusion of other species, thereby decreasing species diversity [27]. An increase in density may most likely be accompanied by an increase in plant cover (e.g., [28]), the latter of which may be influenced directly by an increase in species richness [29]. This is because species diversity and plant density are influenced by immigration intensity: as these two entities increase with immigration (provided the latter is not offset by species’ competitive abilities), site or space occupation within a community increases, and this can conceivably be accompanied by increased plant cover [26]. Conversely, for the effect of plant cover on diversity, plant diversity has been shown to decline when cover is reduced [30].

Since biodiversity assessments and projections reveal that global biodiversity is declining at an alarming rate [31,32], monitoring of species diversity patterns is undoubtedly vital. In particular, it is crucial to pay close attention to plant diversity patterns at the local scale, because diversity changes at this scale are expected to have serious effects on ecosystem functioning [33,34]. This is especially because relationships between diversity and ecosystem functioning are strongest at the local scale [24], and local biodiversity is thus envisaged to play an important role in safeguarding the proper functioning and resilience of ecosystems and the services they provide [35]. In addition, insights on local diversity patterns can inform effective biodiversity conservation measures, thus assisting in managing this natural resource [36,37]. However, Vellend et al. [34] and Valdez et al. [37] caution that improvements are needed in how local diversity is monitored if global trends in species diversity are to be accurately detected.

According to Pausas and Austin [21], monitoring of biodiversity trends should be conducted in relation to environmental conditions, as this can provide better insight into the effects of biodiversity on ecosystem processes. Proper ecosystem functioning is especially crucial in arid and semi-arid regions such as South Africa, as it ultimately affects the provision of ecosystem services that are vital to these regions [38]. To that end, our study aimed to elucidate interactions between plant diversity, abundance, and the growth forms that characterise grasslands, and to investigate the influence of environmental resources on grassland plant diversity in protected and adjacent unprotected areas in Gauteng Province, South Africa. We attempted to answer the following questions: (1) What are the relationships between plant diversity and the two forms of plant abundance (i.e., density and cover)? (2) Do these relationships vary among the different growth forms? (3) How do soil characteristics influence plant diversity and abundance, and also growth forms under the different land-use types? (4) Do soil characteristics and plant growth forms (i.e., traits) jointly influence plant diversity and abundance in the two land-use types? We denote land-use types as formally protected areas (nature reserves) that harbour wild ungulates and unprotected areas used for livestock grazing.

## 2. Materials and Methods

### 2.1. Study Area

The study was conducted in the Grassland Biome of South Africa, the second-largest biome, covering approximately 28% of the country [39]. The study area comprised three nature reserves in Gauteng Province, namely Abe Bailey Nature Reserve (ANR), Roodeplaat Dam Nature Reserve (RNR), and Suikerbosrand Nature Reserve (SNR). Unprotected areas adjacent to the nature reserves were also included in the study. According to the Köppen–Geiger classification, the Gauteng region has a Cwb climate type, which is a warm-temperate climate with dry winters and warm summers [40]. The daily mean temperatures range from 21.2 °C in summer to 9.7 °C in winter, but the northern parts are generally warmer, with mean annual temperatures of approximately 16 °C to 18 °C, compared to around 14 °C to 16 °C in the southern parts of the province [41]. Annual precipitation varies from approximately 600 mm in the lower-lying areas to around 700 mm in the higher-lying areas, and most of the rain falls in summer between October and March [42]. The soils are varied across the province, but according to the FAO soil classification, the soils at the three study locations are predominantly Leptosol (but also Lixisol at ANR), ranging from loam to sandy clay loam at ANR and RNR, and generally clay loam at SNR [43]. Leptosols range from very shallow soils over hard rock to deeper, very gravelly soils [44].

**Figure 1:**
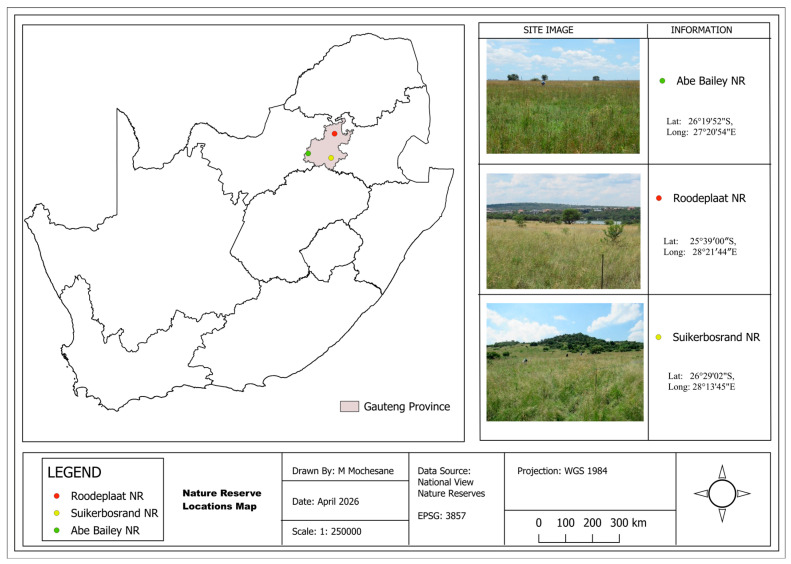
Map of the study area showing the location of the three nature reserves within Gauteng Province.

The three study areas are located within a 100 km radius of Johannesburg, the main industrial and economic region of South Africa. ANR is located south-west of Johannesburg, close to Carletonville town; RNR is to the north, on the outskirts of the city of Tshwane; and SNR is in the south-east, near the town of Heidelberg (Figure 1). Three sites were selected in each of the three protected areas, and each was paired with a corresponding site in an adjacent unprotected area. The selection of sites ensured that paired sites in protected and unprotected areas were close to the border fence demarcating the two land-uses and adjacent to one another, but at least 100 m from the fence. Maximal care was taken to ensure that the paired sites were generally comparable in landscape, topography, and habitats. Two sites at ANR and at SNR were paired with areas on livestock farms; the third site was paired with a communal area generally used for cattle grazing. At RNR, all sites were paired with privately owned smallholdings with very low animal grazing.

### 2.2. Data Collection

Vegetation surveys were conducted during the summer and autumn seasons of 2018, using one Modified Whittaker Plot (MWP) [45] at each site, for a total of 18 MWPs surveyed. At each of the three study locations, there were three MWPs within a protected area, paired with three in the adjacent unprotected areas. Each MWP comprised sample plots of three different sizes: 1 m^2^, 10 m^2^, and 100 m^2^. There were ten sample plots of 1 m^2^ area (2 m × 0.5 m), two plots of 10 m^2^ area (5 m × 2 m), and a single 100 m^2^ plot (20 m × 5 m), and all plots were nested within a single 1000 m^2^ MWP (50 m × 20 m). This manuscript reports data collected in 1 m^2^ plots, totaling 180. We recorded all vascular plant species present in each 1 m^2^ plot and visually estimated their cover using the Braun–Blanquet approach [46,47]. We also determined plant density by counting individuals per species in each 1 m^2^ plot and noted species growth forms, categorising them as grasses, forbs, and shrubs. We collected one soil sample from each 1 m^2^ plot using a 75 mm diameter bucket auger to a depth of 30 cm. A total of 180 soil samples were collected, which were reduced to 54 composite samples, i.e., three composites per 1000 m^2^ MWP. The soil samples were then analysed for calcium (Ca), magnesium (Mg), phosphorus (P), potassium (K), sodium (Na), total nitrogen (TN), organic carbon (OC), total carbon (TC), and pH according to the standard methods compiled by The Non-Affiliated Soil Analysis Work Committee [48]. The methods used were as follows: the Walkley and Black method for OC; the dry combustion method for TC and TN; the Bray method for P; and the soil/water suspension method for soil pH. For the other soil properties (Ca, Mg, K, and Na), the ammonium acetate method was used.

### 2.3. Data Analysis

We analysed plant diversity as species richness (*S*), which we ragrded as the number of species in a 1 m^2^ sample plot. We also included Shannon–Wiener diversity (*H*′) and Pielou evenness (*J*′). However, in this manuscript, we focus on species richness; the details of how *H*′ and *J*′ were determined, along with the study’s findings regarding these indices, are included in the Supplementary Materials. Abundance was measured as the total plant density and cover per 1 m^2^ sample plot. We used SPSS software (version 19) to conduct simple linear regression and Pearson correlation analyses, to determine bivariate relationships between species richness and plant abundance, species richness and growth forms, plant abundance and growth forms, species richness and soil properties, plant abundance and soil properties, and also between growth forms and soil properties. We further used multiple regression to analyse the effects of soil variables on species richness, plant abundance, and growth forms, and also for the combined effects of soil and growth forms on species richness and plant abundance. Our data analysis was performed in two ways: first, for all study sites across the three study locations; and second, separately for each land-use type (protected and unprotected).

Prior to multiple regression, data were transformed and standardised (mean = 0 and standard deviation = 1) to meet the normality assumption. Species richness and soil data were log_10_-transformed, and data for plant density, cover, and growth forms were square root-transformed. We checked for multicollinearity among predictor variables using the variance inflation factor (VIF) values and Pearson correlations. The acceptable VIF limit was set at 10 [49], and the acceptable Pearson correlation coefficient limit was 0.7, such that less than 50% of the variation in each variable was accounted for by its relationship with the counterpart variable [50,51]. Forb density in protected areas and in all study sites, as well as shrub density and forb cover in unprotected areas, showed strong correlations with some of the growth form variables and did not meet the VIF threshold; they were removed from the multivariate analyses. Similarly, most soil variables in the two land-use types were highly correlated and had inflated VIFs; they were omitted, leaving pH, TN, Na, and P for multiple regression analyses.

We assessed species richness and plant abundance patterns among our study sites and locations by conducting Detrended Correspondence Analysis (DCA). We analyzed the three variables as follows: for plant abundance, we used density and cover data; for species richness, we used species presence/absence data as a proxy for richness at each site. DCA is an unconstrained ordination strategy that assessed variation in richness, cover, and density; its advantage was that it provided a summarised visual representation of the richness-abundance relationships between the study sites. We then assessed the responses of species richness, plant density, and plant cover to the soil nutrient gradient by conducting simple linear regressions of the species scores (axes 1 and 2) for these three response variables with the soil variables.

We further compared the responses of species richness, plant density, and cover to soil nutrients using redundancy analysis (RDA). In this analysis, all three variables were included in a single ordination plot as response variables, with soil properties as explanatory variables. We achieved this by using species richness, plant density, and plant cover data for each site rather than individual species (sensu [52]), because CANOCO allows any data to be used whose response we want to predict [53]. We also used this approach to compare the responses of growth form richness, density, and cover to soil nutrients. Both RDA and DCA were conducted using the CANOCO for Windows 4.5 programme [54].

## 3. Results

### 3.1. Patterns of Plant Abundance and Diversity

Average richness, density, and cover across all study sites were 6.72 ± 2.45 (mean ± standard deviation), 29.67 ± 15.26, and 64.30 ± 34.76, respectively. Between the land-use types, species richness averaged 6.69 ± 2.52 in protected areas and 6.75 ± 2.39 in unprotected areas. There was an average plant density of 29.94 ± 17.05 per sample plot in protected areas and 29.40 ± 13.32 in unprotected areas. The average plant cover per sample plot was 66 ± 38% in protected areas and 63 ± 31% in unprotected areas. The two measures of species abundance (i.e., plant cover and density) were positively and significantly correlated for all study sites and also when we distinguished the land-use types (Table 1). Furthermore, density and cover correlated significantly and positively with species richness (*S*) at all study sites and across both land-use types, except that cover was not significantly correlated with species richness in unprotected areas.

### 3.2. Interrelations Among Growth Forms

When we differentiated species into growth forms, the grass components were, in general, positively and significantly correlated with one another across all study sites and in both land-use types (Table S1). Grass richness correlated most strongly with grass density (*r* = 0.481, *p* < 0.001), while grass density correlated strongly with grass cover (*r* = 0.553, *p* < 0.001). Correlations of grass components with forb and shrub components were negative and significant across all study sites; they were significant in protected areas but weak and mostly not statistically significant in unprotected areas. The forb components correlated strongly with each other, and these correlations were equally robust when we differentiated the land-use types. Forb richness correlated strongly with forb density (*r* = 0.627, *p* < 0.001) and with forb cover (*r* = 0.557, *p* < 0.001). The strongest correlation for forb density was with forb cover (*r* = 0.839, *p* < 0.001). There were similarly strong correlations among shrub components across all study sites, which were also evident in both protected and unprotected areas. Shrub richness correlated strongly with shrub density (*r* = 0.701, *p* < 0.001) and with shrub cover (*r* = 0.553, *p* < 0.001). There was also a strong correlation between shrub density and shrub cover (*r* = 0.588, *p* < 0.001). The shrub components correlated mostly positively and significantly with forb components, but between land-use types, significant correlations were only evident in protected areas.

### 3.3. Relationships of Plant Diversity and Abundance with Growth Forms

**Figure 2:**
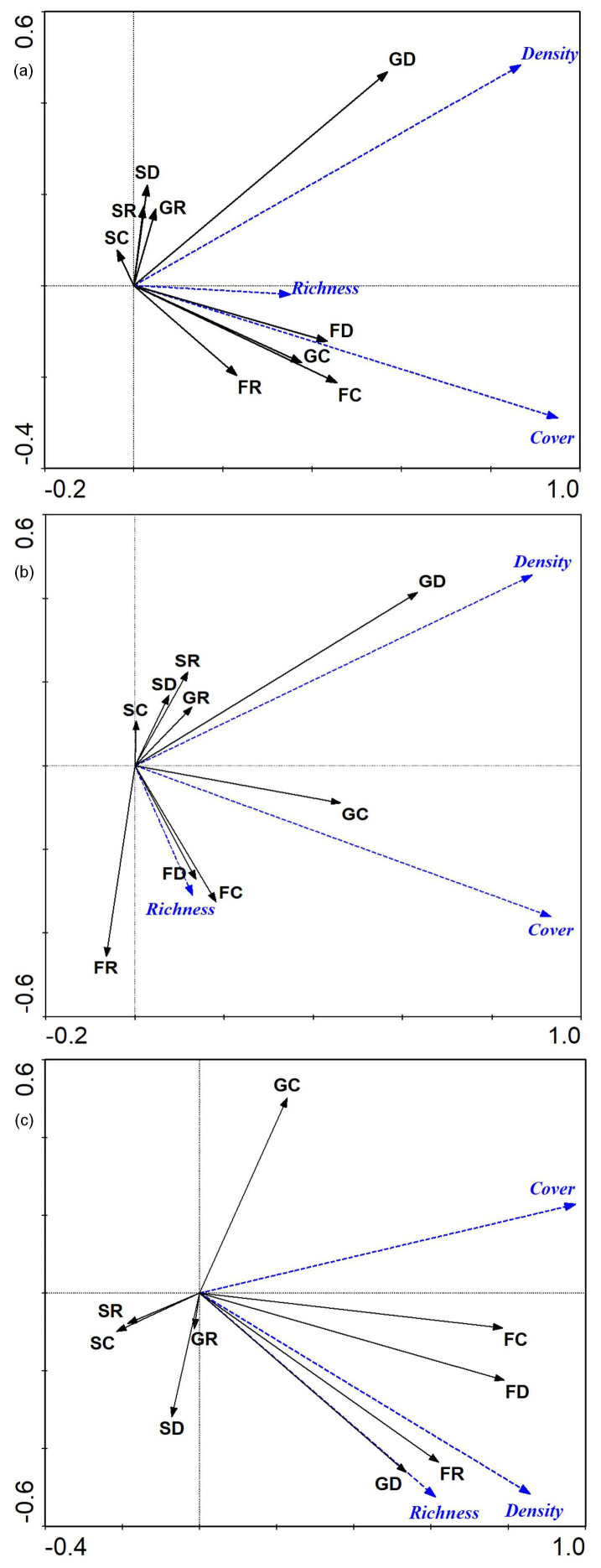
Redundancy analysis (RDA) plots for the first two ordination axes showing the association of growth forms with: (**a**) species richness, plant density, and plant cover for all study sites; (**b**) for protected areas; and (**c**) for unprotected areas. GR is grass richness, GD is grass density, GC is grass cover, FR is forb richness, FD is forb density, FC is forb cover, SR is shrub richness, SD is shrub density, and SC is shrub cover.

Plant density and cover correlated positively and significantly with the richness, density, and cover of grasses and forbs across all study sites, and these strong correlations were also evident when we distinguished protected and unprotected areas (Table 2). The correlations, however, were poor for the shrub components. Total species richness (*S*) showed positive, statistically significant correlations with the richness of grasses, forbs, and shrubs. With regard to the density and cover of the growth forms, *S* only had positive and significant relationships with forb density and cover. Meanwhile, the relations of *S* with grasses and shrubs were generally weak, including in both land-use types. These correlation patterns were partially corroborated by RDA, which showed that species richness and plant cover were mainly associated with the forb components, while plant density was associated with the grass components (Figure 2a). Similar patterns were observed for the protected areas (Figure 2b), but the associations were quite different for the unprotected areas (Figure 2c).

**Figure 3:**
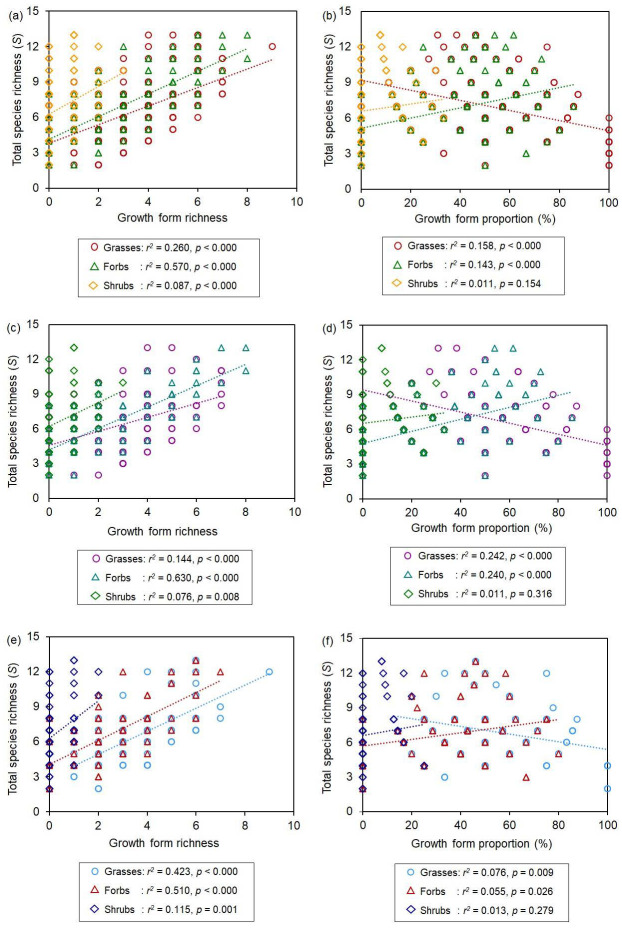
The relationships of total species richness with growth form richness and proportion (i.e., growth form relative richness) for all study sites (**a**,**b**), protected areas (**c**,**d**), and unprotected areas (**e**,**f**). The colours of the dashed lines correspond to the colour codes for grasses, forbs, and shrubs.

Among the growth forms, the strongest predictor of *S* was forb richness, which accounted for more than 50% of the variation in total species richness across all study sites and in both land-use types (Figure 3a,c,e). When we considered the relative or proportional richness among the growth forms, there was a positive relationship between *S* and the increasing proportion of forb species. However, *S* decreased significantly as the proportion of grasses increased (Figure 3b,d,f). The proportion of shrubs did not significantly influence total richness (*S*). Multiple regression showed that, jointly, the growth forms significantly predicted species richness, plant density, and plant cover at all study sites and across land-use types (Table 3).

### 3.4. Effects of Soil Properties on Plant Abundance and Diversity

Species richness showed significant positive relationships with most soil variables, except Na, P, and pH, which showed relatively poor relationships (Figure S1). Among land-use types, the strongest and most significant relationships for species richness were in the unprotected areas and with OC, TC, TN, K, Ca, and Mg. The relationships between plant density and soil variables followed a pattern similar to that of species richness, particularly in the unprotected areas, but plant cover showed no significant correlation with soil nutrients (Table 4). When we considered the four soil variables jointly, only plant density was significantly predicted across all study sites and in both land-use types, with species richness predicted significantly only in the protected areas (Table 3). Plant cover, on the other hand, showed no significant response.

**Figure 4:**
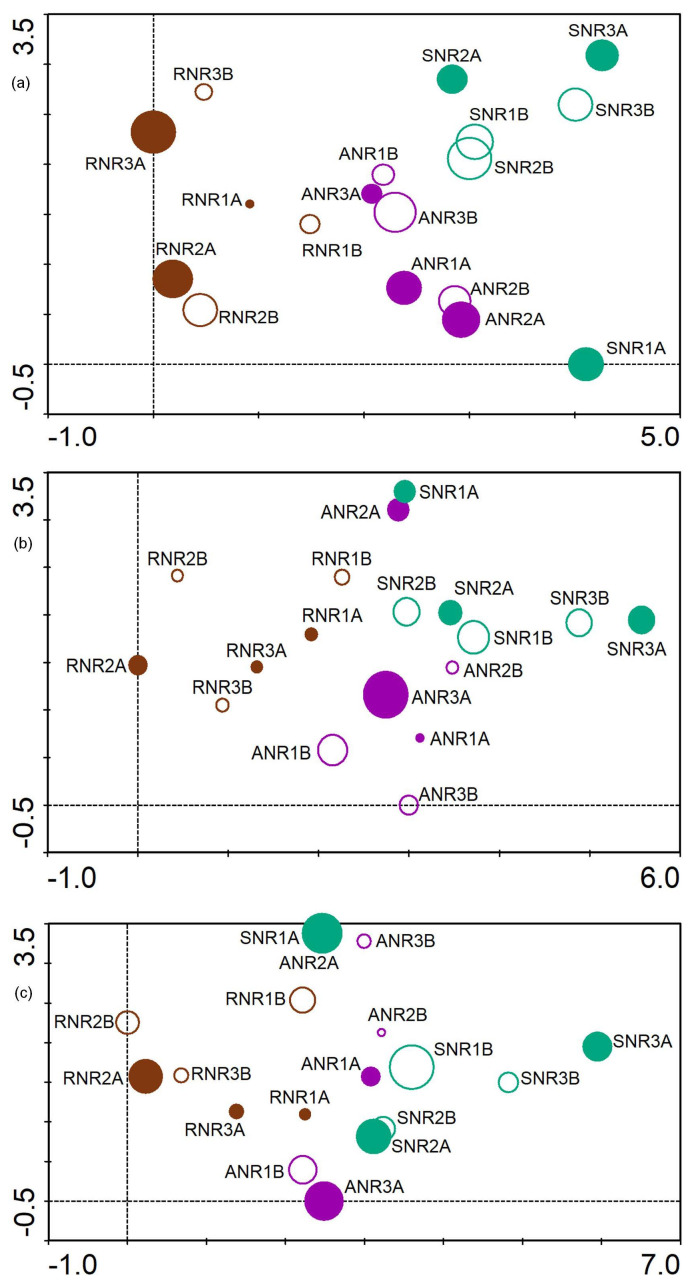
Attribute plots based on detrended correspondence analysis (DCA) for species richness (**a**), plant density (**b**), and plant cover (**c**). ANR, RNR, and SNR denote Abe Bailey, Roodeplaat, and Suikerbosrand study locations, respectively. 1, 2, and 3 denote the three study sites per location. A and B denote protected and unprotected sites, respectively. The sizes of circles correspond to total richness, density, and cover per site.

Across all study sites, without distinguishing land-use types, DCA attribute plots showed variation in species richness, plant density, and plant cover among the study sites. The distribution of sites indicated that, in general, species richness was uniform along both axes 1 and 2 (Figure 4a). Conversely, high plant density and cover (Figure 4b,c) were observed at sites in the right half of the ordination plots along axis 1. Linear regression of the species scores of these three response variables with the soil variables showed a positive response along a soil nutrient gradient on the first two axes (Table 5). The distribution of study sites in the ordination space was associated with increasing soil nutrients for density and cover along axis 1. However, for richness, both axes 1 and 2 showed positive associations with soil variables, except for the negative association of P on axis 2.

**Figure 5:**
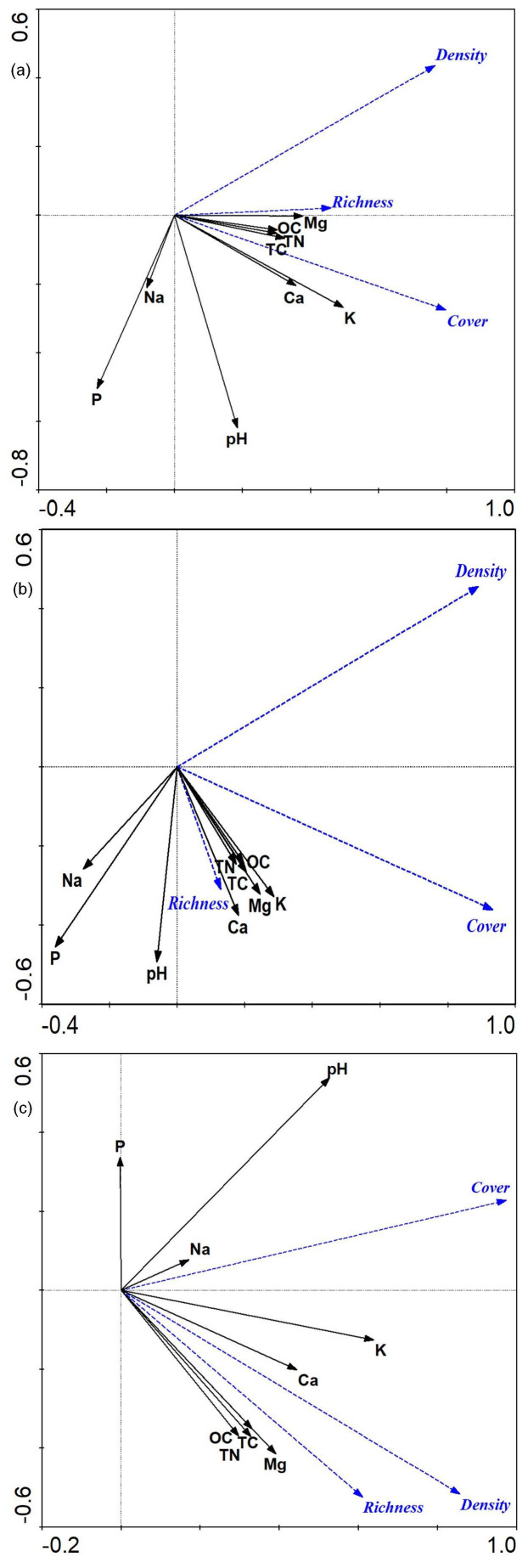
Redundancy analysis (RDA) plots for the first two ordination axes showing the association of soil properties with: (**a**) species richness, plant density, and plant cover for all study sites; (**b**) for protected areas; and (**c**) for unprotected areas.

RDA provided overall visual comparisons of the responses of richness, density, and cover to soil variables and showed a negative association of plant density with P and Na (Figure 5a). On the other hand, richness and cover associated positively with Mg, OC, TN, TC, K, and Ca. These patterns differed when we distinguished land-use types, especially in unprotected areas, where the positive association of Na, pH, and P with plant cover was now evident. Meanwhile, richness and density were positively associated with the remaining soil variables (Figure 5b,c).

### 3.5. Combined Effects of Growth Forms and Soil Variables on Plant Diversity and Abundance

According to multiple regression analysis, the combined influence of growth forms and soil variables was strong and highly significant, accounting for more than 85% of the variance in species richness, plant density, and plant cover at all study sites and in both protected and unprotected areas (Table 3). Grass richness and cover, as well as forb richness, contributed significantly to the species richness model across all study sites (Table 6). For the plant density model, the important variables were grass and shrub density, forb cover, and Na. Meanwhile, for the plant cover model, the important variables were grass and forb cover, shrub density, Na, and P.

### 3.6. Influence of Soil Variables on Plant Growth Forms

**Figure 6:**
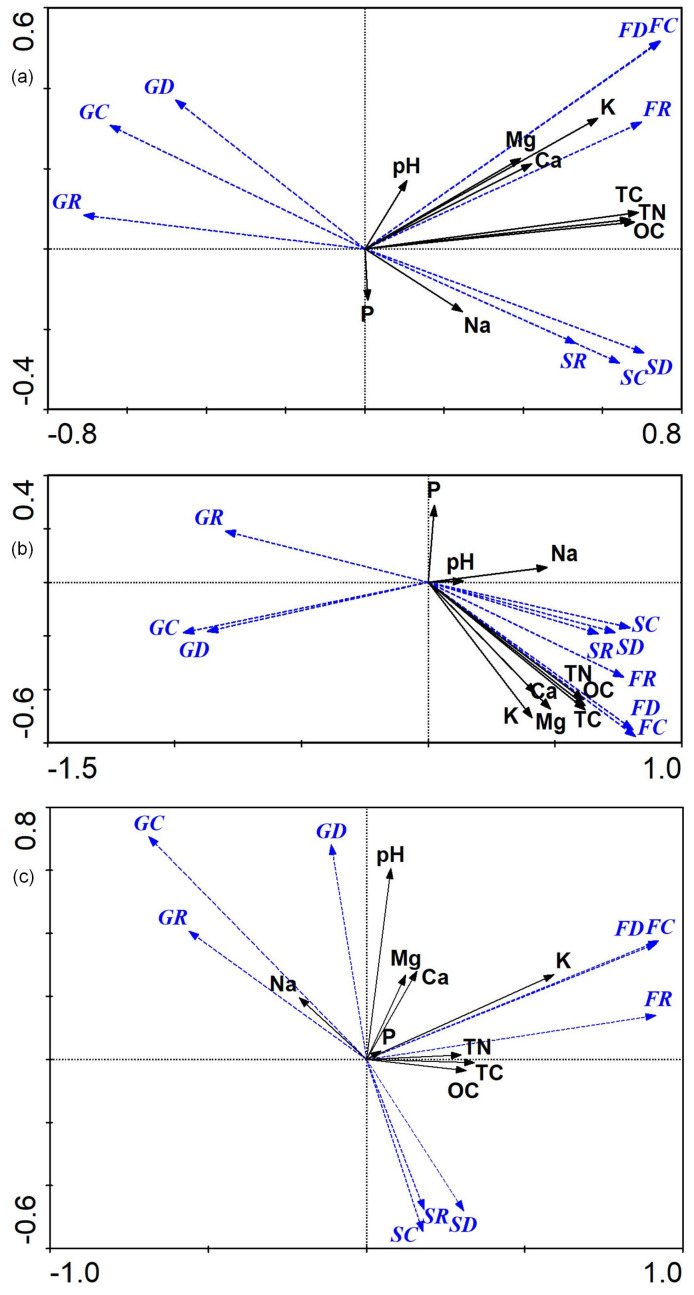
Redundancy analysis (RDA) plots for the first two ordination axes showing the association of soil properties with: (**a**) growth forms for all study sites; (**b**) for protected areas; and (**c**) for unprotected areas. GR is grass richness, GD is grass density, GC is grass cover, FR is forb richness, FD is forb density, FC is forb cover, SR is shrub richness, SD is shrub density, and SC is shrub cover.

Growth forms correlated strongly with the soil variables, particularly with OC, TC, TN, K, Ca, and Mg, and these relations were especially robust in protected areas (Table S2). In the unprotected areas, only forb richness and shrub density showed strong, significant correlations with the soil variables, particularly OC, TC, TN, and K. In general, grasses correlated negatively with soil variables, whereas forbs and shrubs correlated mostly positively with them in both protected and unprotected areas. These patterns were further corroborated by RDA, which showed negative associations between grass components and the soil variables at all study sites and in protected areas, while these soil variables associated positively with the forb and shrub components (Figure 6a,b). For the unprotected areas, the association of growth forms with soil variables was quite different, for instance, with grass components positively associated with Na (Figure 6c). For the combined effects of the soil variables, the strongest associations were with forb richness, density, and cover (Table 7).

## 4. Discussion

### 4.1. Patterns of Plant Diversity Response to Soil Nutrients

Plant diversity is modulated by the availability of environmental resources that regulate plant growth [14], which include water, soil nutrients, and light. Interactions between species diversity and nutrient availability are varied, and studies have found that the relationships are either positive or negative [20]. Species richness has been reported to associate positively with calcium, potassium, magnesium, and phosphorus [17,18], and negatively with organic C and total N [17], and also with phosphorus [19]. However, the hump-shaped pattern is regarded as the typical species diversity response to nutrient availability [21]. Humped-back curves of species richness have been reported for nitrogen, potassium, and phosphorus [16]. In our study, species richness interactions with soil properties were mainly positive (Figure S1).

Patterns in species richness responses to soil nutrients can be explained by Grime’s model [55], which depicts species richness responses to environmental stress. This model is synonymous with the much-reported humped-back curve. According to the model, conditions of low environmental stress (e.g., high soil nutrient levels) are characterised by low species richness because they allow competitive species to flourish and become dominant, thereby excluding less competitive species. Maximum species richness can instead be attained at intermediate levels of environmental stress (e.g., conditions of adequate or optimal soil nutrient levels) because, under such conditions, less competitive species can survive and dominance is reduced. However, as environmental stress increases (e.g., soil nutrient deficiencies), only species tolerant of environmental extremities can flourish [55]. The nutrient levels in our study area were on the lower end of the humped model compared to other studies (e.g., [16,18]), and hence the association of nutrients with species richness was mainly positive. In contrast, studies involving high nutrient levels, such as nutrient enrichment experiments, have reported declining species richness with increasing nutrient levels (e.g., [56]).

Although species richness in our study showed no significant association with soil pH, it is still pertinent to consider the possible indirect influence of pH on the observed species richness patterns. Soil pH, although not a nutrient per se, is important in determining the availability of soil nutrients to plants [57]. Our three study locations were on acidic soils with average pH ranging from 4.84 to 6.82, and the acidic nature of these soils may have affected the availability of some nutrients in our study area. Even though the nutrient levels in our study were relatively higher than those reported in strongly acidic soils (e.g., [58]), they were lower than those reported elsewhere (e.g., [18]). For example, the nitrogen levels we recorded were below the optimum reported by Janssens et al. [16]. This might have been because the pH range favourable for microbial nitrogen fixation and mineralisation is between 6 and 8, and thus the range where nitrogen availability is the highest [57]. For phosphorus availability, the most favourable pH is near-neutral to slightly acidic, with the highest availability in the 6.5–7.0 range [59]. The pH of the soils in our study area ranged mainly below 6.5, a level at which phosphorus becomes insoluble and thus unavailable to plants [57]. As a result, phosphorus is often deficient in acidic soils [60]. The availability of potassium, calcium, and magnesium also decreases with increasing soil acidity [61].

### 4.2. Relations Between Growth Form Richness, Abundance, and Soil Properties

Plant diversity, density, and cover are important plant community characteristics that are interrelated, and they generally exhibit a positive association [23,29,62]. Likewise, we have reported positive correlations in our study area (Table 1). However, these positive relations were not consistent across growth forms upon decoupling species, because we subsequently detected negative correlations between grass richness, density, and cover and the forb and shrub components (Table S1). It thus infers that variation in plant diversity and abundance (i.e., density and cover) may, in part, be modulated by species’ growth forms within a community (Table 2). The reason is that growth forms exhibit plant morphological and physiological strategies for resource utilisation [10]. Species differ in their ability to acquire and utilise nutrient resources, and this ability is strongly influenced by specific plant functional traits linked to nutrient availability, such as root length and specific leaf area [63]. Within similar growth forms, plants may be similarly influenced by soil nutrient availability, but the influence might differ between growth forms [10]. Furthermore, plant species of the same growth form presumably consume similar resources and, accordingly, may share certain morphological and physiological traits linked to resource utilisation, thereby intensifying interspecific competition within growth forms [10,21]. This may lead to low abundance, whereas species that are morphologically dissimilar to neighbours will experience less competition and may have higher abundance [64].

The stochastic niche theory is often invoked to explain patterns of species abundance in communities. According to the theory, there is a correlation between a species’ traits and its abundance, and abundance patterns are largely shaped by competition for environmental resources, such as soil nutrients [65]. Our findings are consistent with this theory because we detected strong, statistically significant correlations between the abundance (i.e., density and cover) of the different growth forms and soil chemical properties (Table S2). The correlation patterns, however, were contrasting between the growth forms: grasses correlated negatively with soil properties, while forbs and shrubs correlated positively. These divergent patterns may be explained by the differing traits of the growth forms. For instance, different growth forms have different rooting depths [2,66] and root systems [67]. In grasslands, most grass species have similar root types concentrated in the upper soil profile, whereas those of forb and woody species vary widely in type and depth distribution [2]. These differences in rooting systems influence the ability of growth forms to utilise resources and may reduce competition for resources or enhance resource partitioning [68]. This is a form of niche differentiation among the different growth forms, and it begets differential use of soil resources, influences the relative abundance of the growth forms, and consequently allows for species coexistence [2].

Even though competition for resources and the resultant exclusion of less competitive species are among the major forms of species interactions, many species are able to coexist in nature. This coexistence is mainly determined by trade-offs between competing species, which pertain to how species allocate resources; these may include differences in resource utilisation rates or in biomass allocation to roots, stems, and leaves [69]. However, species competitive interactions and coexistence are strongly influenced by the traits of dominant species, and thus dominant species are important in shaping the diversity and abundance patterns within communities [70]. This is because morphological and physiological traits of dominant species effect superiority in competition for environmental resources [65,71]. Grasses are generally the dominant growth form in grasslands, and they have the competitive advantage of allocating more biomass to roots, which can give them greater access to nutrients than competitors [2,72]. According to Tilman and Wedin [72], species that have a competitive advantage in resource uptake increase in abundance because they can outcompete other species by lowering the availability of soil nutrients. In our study, the cover and density of grasses showed a negative correlation with soil nutrients, suggesting that under conditions of low nutrient availability, grasses outcompete forbs and shrubs and maintain relatively high abundance. As soil nutrient levels increase, grass dominance decreases, and accordingly, forb and shrub abundance increase. This latter scenario is evidenced by the positive association of forb and shrub density and cover with soil properties (Table S2).

The stochastic niche theory may also be invoked to explain the interrelations among the growth forms in our study. This theory predicts that species that are already established within a community are more likely to inhibit the establishment of newly arriving species that are more similar to them, for instance, those of similar growth form or functional type, and this favourably enhances the abundance of the best competitors [65]. This recruitment limitation can partially suggest that a positive relationship will exist between total species richness and growth form richness. This is evident in our study, as we detected an increase in total species richness with increasing richness across all growth forms (Figure 3a,c,e). However, this pattern differed when we considered the proportional or relative richness of each growth form, as total species richness decreased with increasing proportion of grasses (Figure 3b,d,f). As mentioned earlier, these richness and abundance patterns could result from competition for soil resources between the different growth forms, and the subsequent exclusion of less competitive species. The resources may not only be soil nutrients but also space within a community. In a nutshell, the decline in total species richness with increasing grass proportion that we observed in our study could signify competitive exclusion of forbs and shrubs by grasses.

There are also land-use effects to consider, which are mainly presented as grazing disturbance. In general, grazing exclusion treatments have consistently shown that plant abundance declines with grazing (e.g., [73,74,75]). The effects of herbivory, however, can vary with growth forms (e.g., [76,77]) because, for example, as herbivores consume grasses, they may reduce grass abundance while allowing forbs to increase [2], and shrub abundance may also increase if grazing intensity is high [78]. These herbivory dynamics can thus affect the relations among grasses, forbs, and shrubs. They could even be one of the explanatory mechanisms behind the negative relations we observed between grasses and the other growth forms. However, even though our study was observational and did not determine the causal mechanisms behind these relations, it is still possible to infer probable causation. Our interpretation is that the negative relationships between grasses and other growth forms were primarily due to resource competition rather than herbivory, mainly because grasses remained the dominant growth form in our study area despite herbivore consumption. The reason is that grasses, especially those in grasslands with a long evolutionary history of grazing, such as in Africa, are tolerant of grazing and can recover, sometimes even growing larger than ungrazed plants, a phenomenon termed overcompensation [2]. The grazing effects also vary with herbivore type [74,79], which is the main distinction in our study area. This difference between the two land-use types primarily stems from the dietary preferences of the herbivores involved. It can, therefore, be expected to affect the abundance of the different growth forms differently. However, these impact variations appear minimal, at least with respect to correlations among the growth forms, as they were consistent across both land-use types.

## 5. Conclusions

Patterns of plant diversity at the local scale are mediated by the availability and variability of environmental resources, including soil nutrients. Our findings have indicated that species richness and plant abundance responded mostly positively to soil nutrients in both protected and unprotected areas. Interpretations of species diversity responses to nutrient availability, however, should consider species’ growth forms, as interactions with nutrient resources are tied to traits that differ across growth forms. The importance of uncoupling growth forms was clearly evident in our study. For instance, although we detected positive associations of species richness and abundance with soil nutrients, the dynamics of interaction changed when we differentiated growth forms. The correlations changed from relatively weak and statistically non-significant in protected areas, to becoming more robust and significant when we partitioned species into growth forms. Similarly, the positive relationships of growth forms with species richness and abundance varied upon differentiation of growth forms. All in all, these nuanced relations between plant diversity, abundance, and soil nutrients indicate that decoupling growth forms can provide better insight into diversity and abundance patterns and the potential mechanisms underlying them, because different growth forms respond differently to variation in environmental conditions and resources.

## Figures and Tables

**Table 1:** Model summaries for simple linear regression analysis of species richness, plant density, and plant cover. *n* = 180 for all study sites; df1 = 1, df2 = 178. *n* = 90 for protected areas and also for unprotected areas; df1 = 1, df2 = 88.

Response Variable	Explanatory Variable	*r*	*r* ^2^	ANOVA	
				*F*	*p*
All locations					
Species richness	Plant density	0.408	0.166	35.539	0.000
Species richness	Plant cover	0.225	0.051	9.486	0.002
Plant density/cover	Plant cover/density	0.486	0.237	55.175	0.000
Protected areas					
Species richness	Plant density	0.326	0.106	10.458	0.002
Species richness	Plant cover	0.271	0.073	6.978	0.010
Plant density/cover	Plant cover/density	0.425	0.181	19.447	0.000
Unprotected areas					
Species richness	Plant density	0.524	0.274	33.293	0.000
Species richness	Plant cover	0.168	0.028	2.560	0.113
Plant density/cover	Plant cover/density	0.583	0.340	45.399	0.000

**Table 2:** Pearson correlations of species richness, plant density, and plant cover with growth forms. *n* = 180 for all study sites, *n* = 90 for protected areas, *n* = 90 for unprotected areas.

	Species Richness			Plant Density			Plant Cover		
	All	A	B	All	A	B	All	A	B
GR	0.509 ***	0.380 ***	0.651 ***	0.292 ***	0.301 **	0.294 **	0.111	0.130	0.102
GD	0.112	0.029	0.264 *	0.742 ***	0.802 ***	0.633 ***	0.352 ***	0.299 **	0.471 ***
GC	0.006	0.053	−0.051	0.293 ***	0.326 **	0.242 *	0.796 ***	0.804 ***	0.784 ***
FR	0.755 ***	0.794 ***	0.714 ***	0.249 **	0.136	0.436 ***	0.195 **	0.241 *	0.119
FD	0.448 ***	0.524 ***	0.382 ***	0.484 ***	0.364 ***	0.637 ***	0.280 ***	0.266 *	0.306 **
FC	0.356 ***	0.388 ***	0.321 **	0.386 ***	0.260 *	0.560 ***	0.480 ***	0.521 ***	0.428 ***
SR	0.296 ***	0.276 **	0.339 **	0.087	0.065	0.120	−0.005	−0.067	0.085
SD	0.171 *	0.160	0.188	0.172 *	0.135	0.229 *	0.006	−0.001	0.010
SC	0.102	0.099	0.131	0.016	−0.002	0.057	0.075	0.087	0.033

All: all study sites; A: protected areas; B: unprotected areas; GR: Grass richness; GD: Grass density; GC: Grass cover; FR: Forb richness; FD: Forb density; FC: Forb cover; SR: Shrub richness; SD: Shrub density; SC: Shrub cover; Significance levels: * *p* < 0.05, ** *p* < 0.01, *** *p* < 0.001.

**Table 3:** Summary of multiple regression analyses with species richness, plant density, and plant cover as dependent variables, growth forms (i.e., GR, GD, GC, FR, FD, FC, SR, SD, SC), and soil properties (i.e., TN, Na, P, pH) as predictor variables. *n* = 54 for all study sites, *n* = 27 for protected areas, *n* = 27 for unprotected areas.

Study Sites	Growth Forms			Soil Properties			Growth Forms and Soil		
	*r* ^2^	*F*	*p*	*r* ^2^	*F*	*p*	*r* ^2^	*F*	*p*
Species richness									
All sites	0.895	47.754	0.000	0.155	2.238	0.078	0.903	31.704	0.000
Protected	0.882	16.880	0.000	0.346	2.916	0.045	0.914	12.326	0.000
Unprotected	0.982	150.351	0.000	0.181	1.214	0.333	0.989	126.443	0.000
Plant density									
All sites	0.894	47.562	0.000	0.290	5.006	0.002	0.912	35.609	0.000
Protected	0.951	43.764	0.000	0.492	5.316	0.004	0.968	35.142	0.000
Unprotected	0.972	94.873	0.000	0.361	3.109	0.036	0.983	79.746	0.000
Plant cover									
All sites	0.954	116.49	0.000	0.122	1.702	0.165	0.961	84.706	0.000
Protected	0.967	65.291	0.000	0.110	0.678	0.614	0.974	43.951	0.000
Unprotected	0.941	43.039	0.000	0.257	1.900	0.146	0.958	31.031	0.000

GR: Grass richness; GD: Grass density; GC: Grass cover; FR: Forb richness; FD: Forb density; FC: Forb cover; SR: Shrub richness; SD: Shrub density; SC: Shrub cover.

**Table 4:** Pearson correlations of species richness, plant density, and plant cover with soil variables. *n* = 54 for all study sites, *n* = 27 for protected areas, *n* = 27 for unprotected areas.

Soil Variables	Species Richness			Plant Density			Plant Cover		
	All	A	B	All	A	B	All	A	B
OC (%)	0.324 *	0.334	0.386 *	0.192	0.034	0.413 *	0.125	0.135	0.094
TC (%)	0.323 *	0.333	0.384 *	0.203	0.036	0.438 *	0.149	0.155	0.127
TN (%)	0.329 *	0.336	0.397 *	0.194	0.016	0.455 *	0.129	0.114	0.136
K (mg kg^−1^)	0.279 *	0.189	0.461 *	0.216	0.026	0.582 **	0.209	0.128	0.346
Ca (mg kg^−1^)	0.307 *	0.204	0.451 *	0.181	−0.024	0.453 *	0.223	0.208	0.247
Mg (mg kg^−1^)	0.443 **	0.300	0.614 ***	0.256	0.039	0.495 **	0.145	0.127	0.180
Na (mg kg^−1^)	0.138	−0.020	0.355	−0.125	−0.364	0.169	0.087	−0.000	0.219
P (mg kg^−1^)	−0.058	0.000	−0.204	−0.292 *	−0.318	−0.267	0.008	−0.013	0.019
pH	0.080	0.154	0.011	0.017	0.078	0.155	0.300 *	0.263	0.356

All: all study sites; A: protected areas; B: unprotected areas; Significance levels: * *p* < 0.05, ** *p* < 0.01, *** *p* < 0.001.

**Table 5:** Correlations between soil variables and species scores for the first two ordination axes of detrended correspondence analysis (DCA), for species richness, plant density, and plant cover data. There were no significant correlations for pH.

	Species Richness		Plant Density		Plant Cover	
Soil Variables	Axis 1		Axis 1		Axis 1	
	*r*	*p*	*r*	*p*	*r*	*p*
OC (%)	0.612	0.007	0.637	0.005	0.662	0.003
TC (%)	0.611	0.007	0.628	0.005	0.653	0.003
TN (%)	0.599	0.009	0.631	0.005	0.661	0.003
K (mg kg^−1^)	0.638	0.004	0.611	0.007	0.602	0.008
Ca (mg kg^−1^)	0.508	0.032	0.479	0.044	0.501	0.034
Mg (mg kg^−1^)	0.578	0.012	0.555	0.017	0.589	0.010
	Axis 2					
Na (mg kg^−1^)	0.521	0.026	-	-	-	-
P (mg kg^−1^)	−0.550	0.018	-	-	-	-

**Table 6:** Summary of growth form and soil variables that contributed significantly to the multiple regression models for species richness, plant density, and plant cover.

Response Variables	Predictor Variables	Beta	Std Error	*t*	*p*
Species richness					
All sites	Forb richness	0.941	0.098	9.610	0.000
	Grass richness	0.560	0.078	7.219	0.000
	Grass cover	−0.203	0.090	−2.263	0.029
Protected	Forb richness	0.945	0.228	4.140	0.001
	Grass cover	−0.601	0.177	−3.397	0.004
	Grass richness	0.442	0.160	2.767	0.015
	Grass density	0.445	0.202	2.203	0.045
	pH	0.267	0.125	2.143	0.050
Unprotected	Forb richness	0.991	0.053	18.549	0.000
	Grass richness	0.600	0.041	14.768	0.000
	Shrub richness	0.244	0.068	3.588	0.003
					
Plant density					
All sites	Grass density	0.995	0.083	12.025	0.000
	Forb cover	0.521	0.089	5.866	0.000
	Shrub density	0.391	0.125	3.137	0.003
	Na	−0.268	0.094	−2.838	0.007
Protected	Grass density	1.341	0.123	10.894	0.000
	Shrub density	0.437	0.129	3.402	0.004
	Forb cover	0.378	0.138	2.750	0.016
Unprotected	Grass density	0.788	0.071	11.139	0.000
	Forb density	0.658	0.070	9.446	0.000
					
Plant cover					
All sites	Grass cover	0.1040	0.057	18.320	0.000
	Forb cover	0.764	0.059	12.921	0.000
	Shrub density	0.216	0.083	2.602	0.013
	Na	−0.134	0.063	−2.134	0.039
	P	−0.106	0.043	−2.487	0.017
Protected	Grass cover	1.168	0.097	12.073	0.000
	Forb cover	0.684	0.123	5.540	0.000
	Shrub density	0.316	0.115	2.741	0.016
Unprotected	Grass cover	1.262	0.121	10.415	0.000
	Forb density	0.795	0.110	7.213	0.000
	Shrub richness	0.326	0.135	2.412	0.029
	Grass density	−0.264	0.112	−2.354	0.033

**Table 7:** Summary of multiple regression analysis with growth forms as dependent variables and Total N, Na-Amm Acet, P-Bray1, and pH(H_2_O) as predictor variables. *n* = 54, df1 = 4, df2 = 49 for all study sites. *n* = 27, df1 = 4, df2 = 22 for protected areas and also for unprotected areas.

	All Study Sites			Protected Areas			Unprotected Areas		
	*r* ^2^	*F*	*p*	*r* ^2^	*F*	*p*	*r* ^2^	*F*	*p*
GR	0.164	2.404	0.062	0.464	4.754	0.006	0.104	0.637	0.642
GD	0.146	2.101	0.095	0.563	7.093	0.001	0.122	0.765	0.559
GC	0.091	1.228	0.311	0.314	2.518	0.070	0.139	0.886	0.489
FR	0.362	6.951	0.000	0.631	9.388	0.000	0.282	2.164	0.107
FD	0.401	8.189	0.000	0.679	11.635	0.000	0.398	3.635	0.020
FC	0.351	6.620	0.000	0.669	11.109	0.000	0.274	2.079	0.118
SR	0.076	1.009	0.412	0.133	0.846	0.511	0.068	0.401	0.806
SD	0.196	2.990	0.028	0.238	1.714	0.183	0.193	1.312	0.296
SC	0.135	1.913	0.123	0.206	1.423	0.259	0.094	0.570	0.687

GR: Grass richness; GD: Grass density; GC: Grass cover; FR: Forb richness; FD: Forb density; FC: Forb cover; SR: Shrub richness; SD: Shrub density; SC: Shrub cover.

## Data Availability

The original contributions presented in this study are included in the article/Supplementary Materials. Further inquiries can be directed to the corresponding author.

## Supplementary Materials

The following supporting information can be downloaded at https://www.mdpi.com/article/10.3390/plants15121895/s1. Figure S1: Relationships between plant diversity and soil chemical properties across all study sites, and also when distinguishing protected and unprotected areas; Table S1: Pearson correlations between growth forms. *n* = 180 for all study sites, *n* = 90 for protected areas, *n* = 90 for unprotected areas; Table S2: Pearson correlations between growth forms and soil variables. *n* = 54 for all study sites, *n* = 27 for protected areas, *n* = 27 for unprotected areas; Table S3: Average (± standard deviation) species richness, plant density, and plant cover per site; Table S4: Soil properties per study site; Table S5: Formulae for determining Shannon-Wiener diversity and Pielou evenness indices; Table S6: Pearson correlations of H’ and J’ with growth forms. *n* = 180 for all study sites, *n* = 90 for protected areas, *n* = 90 for unprotected areas; Table S7: Multiple regression analysis with H’ and J’ indices as dependent variables, growth forms and soil properties as predictor variables. *n* = 54 for all study sites, *n* = 27 for protected areas, and also for unprotected areas; Table S8: Pearson correlations of H’ and J’ indices with soil variables. *n* = 54 for all study sites, *n* = 27 for protected areas, *n* = 27 for unprotected areas.
