# Supplementary material for: Plant Diversity and Abundance Response to the Effects of Soil Properties and Growth Forms in Grasslands
Source: Plants (Basel). 2026 Jun 18;15(12):1895. doi: 10.3390/plants15121895 (PMC13306569; doi:10.3390/plants15121895)
Supplement: Supplementary file 1 [file plants-15-01895-s001.zip › plants-4232325 Supplementary Materials.pdf]

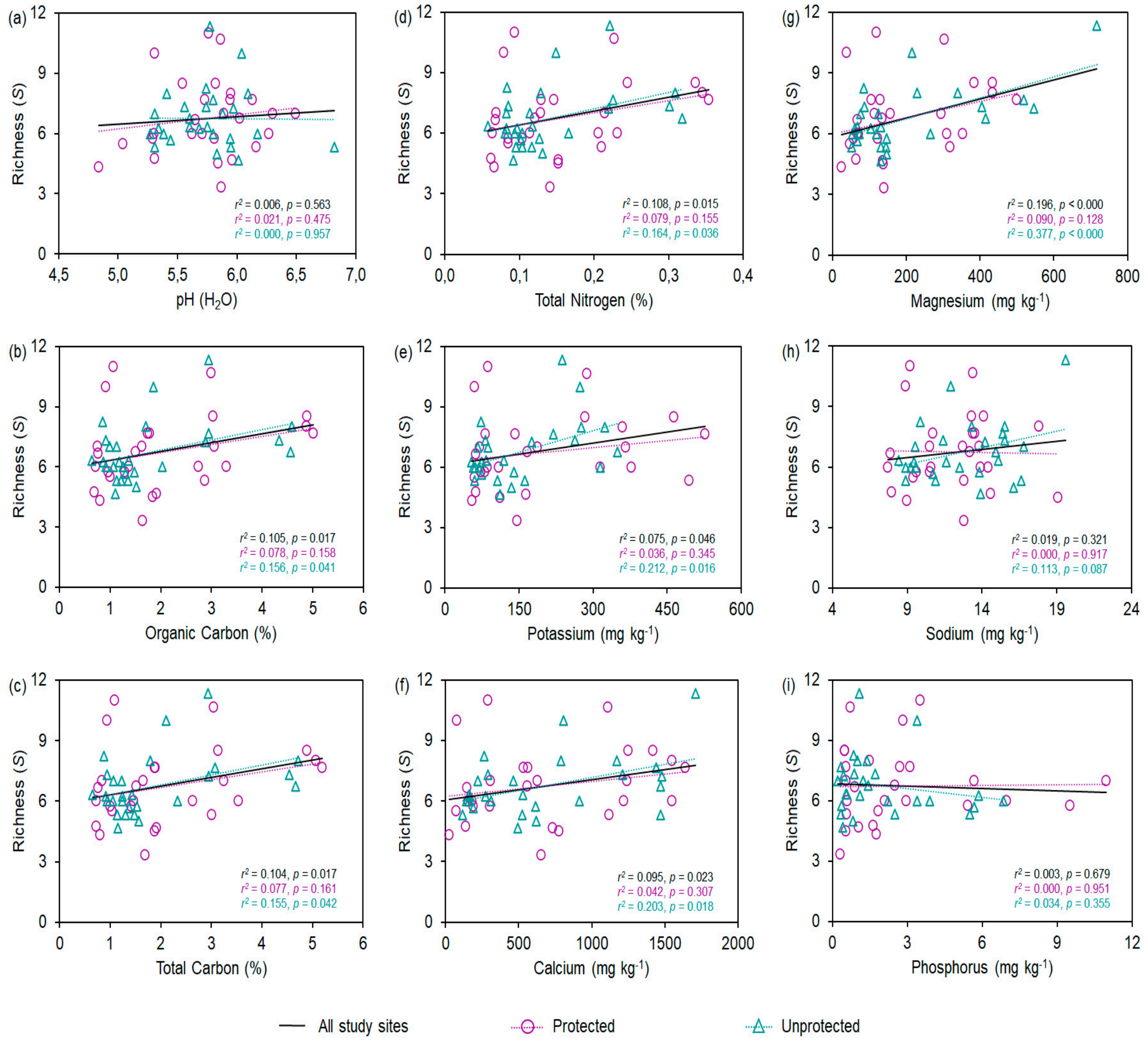

Figure S1. Relationships between plant diversity and soil chemical properties across all study sites, and also when distinguishing protected and unprotected areas.

Table S1. Pearson correlations between growth forms. n = 180 for all study sites, n = 90 for protected areas, n = 90 for unprotected areas.

Growth forms	GR	GD	GC	FR	FD	FC	SR	SD
				All study sites				
GD	0.481***							
GC	0.295***	0.553***						
FR	-0.126	-0.191*	-0.166*					
FD	-0.137	-0.184*	-0.245**	0.627***				
FC	-0.203**	-0.195**	-0.130	0.557***	0.839***			
SR	-0.166*	-0.200**	-0.221**	0.196**	0.172*	0.196**		
SD	-0.261***	-0.229**	-0.264***	0.210**	0.241**	0.273***	0.701***	
SC	-0.248**	-0.214**	-0.176*	0.159*	0.119	0.150*	0.553***	0.588***
				Protected Areas				
GD	0.529***							
GC	0.393***	0.527***						
FR	-0.201	-0.277**	-0.122					
FD	-0.267*	-0.227*	-0.216*	0.713***				
FC	-0.294**	-0.230*	-0.063	0.583***	0.780***			
SR	-0.291**	-0.245*	-0.330**	0.217*	0.286**	0.262*		
SD	-0.373***	-0.295**	-0.364***	0.235*	0.391***	0.425***	0.710***	
SC	-0.339**	-0.240*	-0.235*	0.209*	0.218*	0.234*	0.489***	0.542***
				Unprotected Areas				
GD	0.447***							
GC	0.189	0.624***						
FR	-0.023	-0.010	-0.235*					
FD	-0.026	-0.144	-0.280**	0.556***				
FC	-0.104	-0.147	-0.217*	0.528***	0.901***			
SR	0.031	-0.112	-0.059	0.153	0.052	0.102		
SD	-0.114	-0.106	-0.125	0.169	0.091	0.079	0.686***	
SC	-0.071	-0.144	-0.064	0.029	-0.024	-0.013	0.741***	0.754***

GR: Grass richness; GD: Grass density; GC: Grass cover; FR: Forb richness; FD: Forb density; FC: Forb cover; SR: Shrub richness; SD: Shrub density; SC: Shrub cover; Significance levels: * $p < 0.05$, ** $p < 0.01$, *** $p < 0.001$

Table S2. Pearson correlations between growth forms and soil variables. *n* = 54 for all study sites, *n* = 27 for protected areas, *n* = 27 for unprotected areas.

Soil variables	GR	GD	GC	FR	FD	FC	SR	SD	SC
				All study sites					
OC (%)	-0.399**	-0.277*	-0.260	0.529***	0.472***	0.476***	0.523***	0.676***	0.505***
TC (%)	-0.409**	-0.278*	-0.253	0.537***	0.494***	0.503***	0.521***	0.674***	0.507***
TN (%)	-0.383**	-0.273*	-0.255	0.529***	0.479***	0.477***	0.504***	0.660***	0.495***
K (mg kg^{-1})	-0.356**	-0.206	-0.149	0.476***	0.459***	0.476***	0.434**	0.538***	0.345*
Ca (mg kg^{-1})	-0.148	-0.157	-0.059	0.394**	0.362**	0.370**	0.326*	0.442**	0.305*
Mg (mg kg^{-1})	-0.024	-0.088	-0.124	0.459***	0.384**	0.358**	0.264	0.420**	0.257
Na (mg kg^{-1})	-0.087	-0.277*	-0.054	0.189	0.098	0.139	0.312*	0.339*	0.343*
P (mg kg^{-1})	-0.017	-0.216	0.025	-0.022	-0.039	-0.001	-0.198	-0.192	-0.104
pH	-0.007	-0.065	0.168	0.058	0.094	0.200	0..111	0.093	0.052
				Protected Areas					
OC (%)	-0.581**	-0.440*	-0.353	0.589**	0.605**	0.672***	0.655***	0.707***	0.571**
TC (%)	-0.588**	-0.443*	-0.344	0.598**	0.616**	0.688***	0.645***	0.706***	0.576**
TN (%)	-0.563**	-0.448*	-0.363	0.584**	0.594**	0.655***	0.636***	0.699***	0.567**
K (mg kg^{-1})	-0.440*	-0.313	-0.202	0.445*	0.411*	0.467*	0.515**	0.555**	0.353
Ca (mg kg^{-1})	-0.451*	-0.368	-0.179	0.469*	0.435*	0.539**	0.483*	0.541**	0.414*
Mg (mg kg^{-1})	-0.432*	-0.364	-0.275	0.522**	0.497**	0.561**	0.526**	0.628***	0.439*
Na (mg kg^{-1})	-0.446*	-0.522**	-0.268	0.289	0.230	0.326	0.354	0.364	0.462*
P (mg kg^{-1})	0.091	-0.194	0.032	-0.010	-0.073	-0.033	-0.229	-0.193	-0.148
pH	-0.115	-0.142	0.115	0.187	0.028	0.210	0.158	0.231	0.125
				Unprotected Areas					
OC (%)	-0.125	0.057	-0.124	0.445*	0.329	0.238	0.292	0.637***	0.389*
TC (%)	-0.142	0.061	-0.119	0.452*	0.363	0.278	0.305	0.633***	0.390*
TN (%)	-0.102	0.109	-0.086	0.448*	0.350	0.253	0.261	0.599**	0.351
K (mg kg^{-1})	-0.140	0.098	-0.055	0.554**	0.590**	0.524**	0.201	0.483*	0.235
Ca (mg kg^{-1})	0.232	0.264	0.116	0.301	0.287	0.182	0.096	0.305	0.121
Mg (mg kg^{-1})	0.352	0.341	0.041	0.421*	0.296	0.192	-0.004	0.222	0.055
Na (mg kg^{-1})	0.271	0.148	0.244	0.082	-0.031	-0.045	0.321	0.361	0.273
P (mg kg^{-1})	-0.132	-0.309	-0.002	-0.052	0.012	0.041	-0.199	-0.237	-0.090
pH	0.146	0.107	0.258	-0.127	0.172	0.187	0.030	-0.157	-0.131

GR: Grass richness; GD: Grass density; GC: Grass cover; FR: Forb richness; FD: Forb density; FC: Forb cover; SR: Shrub richness; SD: Shrub density; SC: Shrub cover; Significance levels: * $p < 0.05$, ** $p < 0.01$, *** $p < 0.001$.

Table S3. Average (± standard deviation) species richness, plant density, and plant cover per site.

Study site	Species richness	Plant cover	Plant density
ANR1A	7.7 ± 3.47	54.84 ± 46.2	21.7 ± 17.21
ANR1B	6.4 ± 1.78	69 ± 29.62	39.3 ± 14.29
ANR2A	6.5 ± 2.88	54.2 ± 19.35	29.8 ± 9.34
ANR2B	5.8 ± 1.62	45 ± 30.54	20.2 ± 3.99
ANR3A	5.7 ± 1.95	78.2 ± 30.37	55 ± 25.98
ANR3B	7.4 ± 3.20	50.7 ± 27.07	27.4 ± 9.44
RNR1A	4.2 ± 1.23	54.6 ± 17.51	19.8 ± 7.32
RNR1B	5.7 ± 1.7	68.5 ± 45.33	23.1 ± 9.83
RNR2A	6.4 ± 1.78	76.4 ± 66.25	26.7 ± 14.96
RNR2B	5.8 ± 1.81	64.2 ± 25.06	19.5 ± 6.42
RNR3A	7.1 ± 2.64	48.9 ± 30.08	19 ± 10.74
RNR3B	5.8 ± 1.81	56.85 ± 22.81	22.1 ± 7.4
SNR1A	6.1 ± 1.6	87.1 ± 21.69	29.4 ± 6.45
SNR1B	8.0 ± 3.06	87.5 ± 29.2	41.2 ± 17.43
SNR2A	8.4 ± 2.63	77.7 ± 49.41	33 ± 13.23
SNR2B	8.6 ± 2.67	64.61 ± 24.6	36.1 ± 11.76
SNR3A	8.1 ± 1.52	60.1 ± 30.57	35.1 ± 11.6
SNR3B	7.3 ± 1.95	58.17 ± 28.89	35.7 ± 11.94

A: protected areas; B: unprotected areas

Table S4. Soil properties per study site

Site	pH	OC (%)	TC (%)	TN (%)	K (mg kg^{-1})	Ca (mg kg^{-1})	Mg (mg kg^{-1})	Na (mg kg^{-1})	P (mg kg^{-1})
ANR1A	5.82	0.93	0.974	0.082	79.5	300.333	118.667	9.773	7.99
ANR1B	5.597	0.967	1.026	0.092	62.867	268.667	99.733	9.41	3.05
ANR2A	5.0637	0.907	0.927	0.0767	64.067	52.8	37.167	9.093	2.147
ANR2B	5.32	1.147	1.146	0.097	59.133	140.667	59.667	9.123	3.267
ANR3A	5.557	0.723	0.739	0.065	77.967	146.667	66.8	7.847	1.547
ANR3B	5.693	0.8	0.824	0.075	78.933	237.667	80	9.9	1.033
RNR1A	5.89	1.8	1.826	0.148	140.333	719.667	138.333	15.5	0.637
RNR1B	5.84	1.457	1.508	0.125	131.333	588	141.333	15	0.58
RNR2A	5.51	1.463	1.595	0.116	80.067	312	78.067	10.6	3.687
RNR2B	5.867	1.277	1.366	0.105	102.967	609.667	91.033	11.067	5.677
RNR3A	6.08	1.64	1.683	0.132	165	573	140.667	13.1	3.77
RNR3B	5.947	1.103	1.173	0.093	101.433	492.333	129.333	15.767	0.32
SNR1A	6.243	3.073	3.267	0.218	412.667	1303.333	307.667	13.667	2.693
SNR1B	6.1	1.863	2.078	0.148	288	837	237.333	12.567	2.33
SNR2A	5.673	2.923	2.939	0.226	295.667	1193.333	346.667	13.533	0.597
SNR2B	5.847	2.923	2.985	0.222	210.333	1543.333	593.667	16.4	0.627
SNR3A	5.967	4.923	5.051	0.345	450	1536.667	456	15.133	0.84
SNR3B	5.523	4.493	4.641	0.309	311.667	1283.333	386.333	15.3	1.097

A: protected areas; B: unprotected areas

Table S5. Formulae for determining Shannon-Wiener diversity and Pielou evenness indices.

Index	Formula	Definition of components
Shannon-Wiener	$H' = \sum_{i=1}^{s} p_i \, In \, p_i$	*s* is the number of species (richness). *pi* is the number of individuals of the *i*-th species.
Pielou	$J' = \frac{H'}{\text{In}S}$	*H* is the Shannon-Wiener diversity. *S* is the number of species

Table S6. Pearson correlations of *H′* and *J′* with growth forms. *n* = 180 for all study sites, *n* = 90 for protected areas, *n* = 90 for unprotected areas.

	**Shannon-Wiener diversity (*H′*)**			**Pielou evenness (*J′*)**		
	**All**	**A**	**B**	**All**	**A**	**B**
GR	0.434***	0.293**	0.582***	0.122	0.022	0.227*
GD	-0.058	-0.126	0.043	-0.272***	-0.313**	-0.272**
GC	-0.143	-0.102	-0.191	-0.301***	-0.288**	-0.335**
FR	0.648***	0.727***	0.566***	0.177*	0.190	0.171
FD	0.376***	0.510***	0.265*	0.068	0.103	0.047
FC	0.305***	0.419***	0.189	0.037	0.120	-0.033
SR	0.286***	0.266*	0.319**	0.123	0.070	0.159
SD	0.204**	0.205	0.202	0.126	0.123	0.122
SC	0.178*	0.200	0.163	0.139	0.156	0.119

All: all study sites; A: Protected areas; B: Unprotected areas; GR: Grass richness; GD: Grass density; GC: Grass cover; FR: Forb richness; FD: Forb density; FC: Forb cover; SR: Shrub richness; SD: Shrub density; SC: Shrub cover; Significance levels: * $p < 0.05$, ** $p < 0.01$, *** $p < 0.001$

Table S7. Multiple regression analysis with *H′* and *J′* indices as dependent variables, growth forms and soil properties as predictor variables. *n* = 54 for all study sites, *n* = 27 for protected areas, and also for unprotected areas.

**Study sites**	**Growth forms**			**Soil properties**			**Growth forms and soil**		
	r^2	***F***	***p***	r^2	***F***	***p***	r^2	***F***	***P***
				Shannon-Wiener diversity (H′)					
All sites	0.793	21.557	***0.000***	0.139	1.974	0.113	0.806	14.224	***0.000***
Protected	0.861	13.953	***0.000***	0.329	2.701	0.057	0.905	11.124	***0.000***
Unprotected	0.793	10.410	***0.000***	0.179	1.200	0.339	0.823	6.344	***0.001***
				Pielou evenness (J′)					
All sites	0.535	6.484	***0.000***	0.216	3.367	***0.016***	0.635	5.949	***0.000***
Protected	0.632	3.860	***0.008***	0.457	4.622	***0.007***	0.816	5.187	***0.002***
Unprotected	0.518	2.914	***0.030***	0.245	1.782	0.168	0.592	1.979	0.109

Table S8. Pearson correlations of *H'* and *J'* indices with soil variables. *n* = 54 for all study sites, *n* = 27 for protected areas, *n* = 27 for unprotected areas.

Soil variables	Shannon-Wiener diversity (*H'*)			Pielou evenness (*J'*)		
	All	A	B	All	A	B
OC (%)	0.352**	0.375	0.315	0.161	0.294	0.032
TC (%)	0.342*	0.372	0.297	0.140	0.285	0.001
TN (%)	0.351**	0.386*	0.296	0.157	0.328	-0.008
K (mg kg^{-1})	0.242	0.244	0.235	0.015	0.120	-0.167
Ca (mg kg^{-1})	0.273*	0.304	0.233	0.037	0.291	-0.160
Mg (mg kg^{-1})	0.399**	0.390*	0.430*	0.107	0.294	0.010
Na (mg kg^{-1})	0.219	0.182	0.280	0.222	0.509**	0.062
P (mg kg^{-1})	-0.012	0.066	-0.163	0.102	0.158	-0.015
pH	-0.014	0.164	-0.264	-0.164	0.135	-0.430*
